# Exogenous Ketones Lower Post-exercise Acyl-Ghrelin and GLP-1 but Do Not Impact *Ad libitum* Energy Intake

**DOI:** 10.3389/fnut.2020.626480

**Published:** 2021-01-20

**Authors:** Tetsuro E. Okada, Tony Quan, Marc R. Bomhof

**Affiliations:** Department of Kinesiology and Physical Education, University of Lethbridge, Lethbridge, AB, Canada

**Keywords:** ketone monoester, energy intake, appetite, acyl-ghrelin, GLP-1, PYY

## Abstract

Ketosis and exercise are both associated with alterations in perceived appetite and modification of appetite-regulating hormones. This study utilized a ketone ester (*R*)-3-hydroxybutyl (*R*)-3-hydroxybutyrate (KE) to examine the impact of elevated ketone body D-β-hydroxybutyrate (βHB) during and after a bout of exercise on appetite-related hormones, appetite perception, and *ad libitum* energy intake over a 2 h post-exercise period. In a randomized crossover trial, 13 healthy males and females (age: 23.6 ± 2.4 years; body mass index: 25.7 ± 3.2 kg·m^−2^) completed an exercise session @ 70% VO_2peak_ for 60 min on a cycling ergometer and consumed either: (1) Ketone monoester (KET) (0.5 g·kg^−1^ pre-exercise + 0.25 g·kg^−1^ post-exercise); or (2) isocaloric dextrose control (DEX). Transient ketonaemia was achieved with βHB concentrations reaching 5.0 mM (range 4.1–6.1 mM) during the post-exercise period. Relative to the dextrose condition, acyl-ghrelin (*P* = 0.002) and glucagon-like peptide-1 (*P* = 0.038) were both reduced by acute ketosis immediately following exercise. AUC for acyl-ghrelin was lower in KET compared to DEX (*P* = 0.001), however there were no differences in AUC for GLP-1 (*P* = 0.221) or PYY (*P* = 0.654). Perceived appetite (hunger, *P* = 0.388; satisfaction, *P* = 0.082; prospective food consumption, *P* = 0.254; fullness, *P* = 0.282) and 2 h post-exercise *ad libitum* energy intake (*P* = 0.488) were not altered by exogenous ketosis. Although KE modifies homeostatic regulators of appetite, it does not appear that KE acutely alters energy intake during the post-exercise period in healthy adults.

## Introduction

Recently there has been considerable interest in the utilization of ketogenic diets to help individuals control body weight. Although there is no clear evidence that low carbohydrate, ketogenic diets modify energy expenditure, evidence suggests that ketosis is associated with reduced hunger and improved appetite regulation (1). Weight reduction is generally accompanied by an orexigenic response, with prolonged elevations in the hunger hormone ghrelin and reduced satiety hormones leptin and peptide YY (PYY) (2). Elevated concentrations of ghrelin with weight loss are blunted with very low energy and low carbohydrate, ketogenic diets (3). Although the mechanisms by which ketosis elicits anorexigenic effects on regulators of appetite remain unclear, the ketone body d-β-hydroxybutyrate (βHB) is inversely associated with the hunger hormone ghrelin (3). To avoid restricting carbohydrate or energy intake to induce endogenous ketosis, exogenous ketone esters have been utilized to safely and effectively induce a state of acute ketosis (4). Stubbs et al. (5) investigated the impact of a ketone monoester (R)-3-hydroxybutyl (R)-3-hydroxybutyrate (KE), compared to an isocaloric dextrose control, on appetite and found that the acute elevation of blood βHB reduced hunger and acyl-ghrelin for up to 4 h. Research in rodents shows that a diet providing ~30% of energy from ketone esters can suppress overall energy intake (6).

Exercise is another stimulus that transiently modifies homeostatic regulators of appetite. Both moderate and high intensity aerobic exercise sessions acutely reduce acyl-ghrelin and increase satiety hormones glucagon-like peptide-1 (GLP-1) and PYY for a post-exercise period of ~30–90 min (7–10). The impact of ketosis during and after physical activity on homeostatic markers of appetite and energy intake remains to be fully elucidated. Similar to the impact of ketosis under sedentary conditions, ketone ester supplementation, on top of a background intake of 60 g carbohydrate·h^−1^, during an intense and prolonged bout of exercise is associated with reductions in ghrelin and perceptions of hunger in elite athletes (11).

It remains to be determined whether ketosis, relative to an isocaloric control, impacts homeostatic regulators of appetite following a standardized, ecologically-valid exercise session in normal, healthy individuals. Furthermore, studies to date have not examined the influence of acute ketosis on *ad libitum* energy intake. The objective of our study was to examine the impact of ketosis during and after an exercise session, relative to an isocaloric control, on appetite-regulating hormones, *ad libitum* post-exercise energy intake, and perceived appetite in healthy adults.

## Materials and Methods

### Participants and Ethical Approval

Thirteen participants (seven female; six male) were recruited to take part in the study from the University of Lethbridge and surrounding area using posters and word of mouth. Participants were self-reported, weight stable (had not gained or lost more than 2 kg within the previous 3 months) and had no history of serious physical injuries or metabolic disease (e.g., hypertension, cardiovascular disease, or diabetes). Participants provided written informed consent prior to taking part in the study. The study was approved by the University of Lethbridge Human Participant Research Committee and was conducted in accordance with the ethical principles of the 1964 *Declaration of Helsinki*.

### Baseline Testing

Participants were asked to refrain from alcohol, caffeine, and moderate to vigorous exercise for 24 h prior to the baseline testing. Measures of height and weight were recorded to the nearest 0.1 cm and 0.1 kg, respectively, on a mechanical beam scale (Health-o-Meter Professional, McCook, IL, USA). Participants completed an incremental ramp test to exhaustion on an electromagnetically braked cycle ergometer (Velotron, QUARQ, Spearfish, SD, USA) to assess peak oxygen uptake (VO_2peak_). After an initial 5 min, 50-watt warm-up, participants cycled at 50 W for 4-min at 80 rpm, followed by a constant increase in workload (30 W·min^−1^ for males; 25 W·min^−1^ for females), until volitional fatigue. Oxygen consumption was continuously measured using the Quark CPET (COSMED, Chicago, IL, USA) with breath-by-breath analysis. Heart rate was measured by a Garmin heart rate monitor (HRM-Dual, Garmin, Olathe, KS, USA). All participants achieved an RER > 1.1. VO_2peak_ was calculated by the maximum rolling 30-s VO_2_ (mL O_2_·min^−1^·kg^−1^) average.

### Experimental Conditions

**Figure 1:**
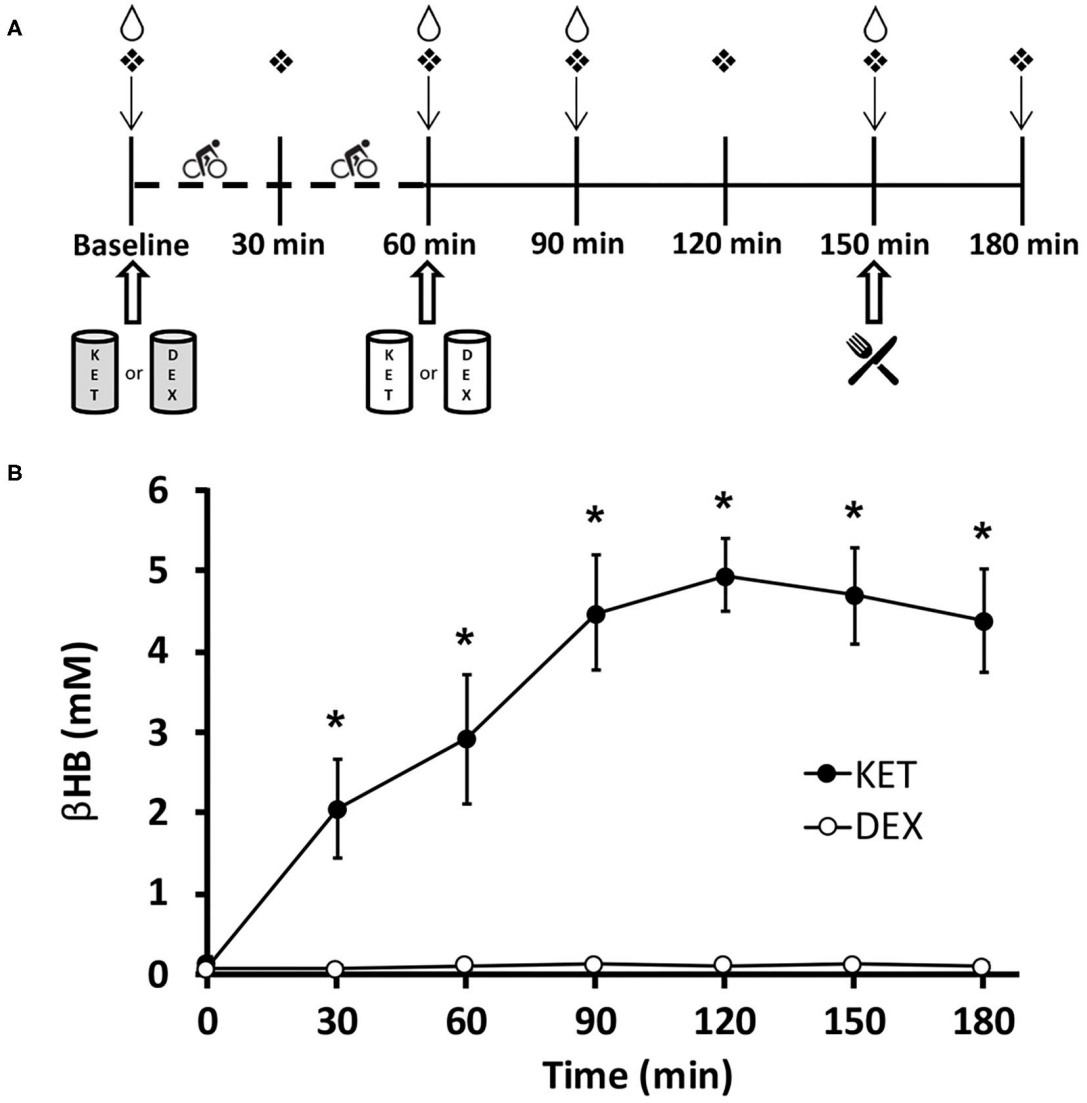
**(A)** Timeline of experimental conditions. Participants arrived at the laboratory in the morning (0800–0900 h) in a fasted state (>10 h). ↓Appetite visual analog scale; 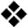 finger capillary draw; 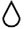 venipuncture; 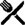 *ad libitum* meal; 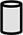 0.5 g·kg^−1^ KET or isocaloric DEX; 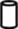 0.25 g·kg^−1^ KET or isocaloric DEX. Dashed timeline represents 1 h of exercise cycling at 70% VO_2_peak. **(B)** Blood d-β-hydroxybutyrate levels in participants for both KET and DEX drink conditions. Values are means ± SD, *n* = 13. *P < 0.05 between KET and DEX conditions. βHB, d-β-hydroxybutyrate; DEX, dextrose; KET, ketone monoester.

In a randomized crossover trial, participants completed a 1-h exercise session at 70% VO_2peak_ and consumed either: (1) ketone monoester drink (KET); or (2) isocaloric dextrose control drink (DEX) (Figure 1A). Six participants started with the DEX trial and seven started with the KET trial. Trials were scheduled at least 1 week apart for males and 4 weeks apart for females. To control for potential appetite fluctuations, all females completed both conditions within the first week of the follicular phase of the menstrual cycle (12, 13).

For each trial, participants reported to the lab in the morning (0800–0900 h) following an overnight fast (>10 h). Prior to each condition, participants were asked to refrain from consuming beverages containing caffeine and alcohol as well-moderate to vigorous exercise for 24 h prior to each trial. Participants were instructed to adhere to similar eating patterns for 24 h prior to the start of each experimental condition, but this was not confirmed using food records or self-reported compliance. Each condition consisted of a 5 min warm-up at 50 W, followed by 60 min at 70% VO_2peak_. During exercise, each participant's oxygen consumption was measured until stable at 70% VO_2peak._ Subsequent spot checks of oxygen consumption were performed every 5 min to ensure consistent exercise intensity. Prior to the initiation of exercise, participants consumed 0.5 g·kg^−1^ body weight KE or an isocaloric amount of dextrose. Given that ketone bodies are oxidized at a greater rate during exercise (14), we provided an additional 0.25 g·kg^−1^ KE post-exercise or isocaloric amount of dextrose in the KET and DEX groups, respectively. The KET beverage was prepared using the commercially available ketone monoester (*R*)-3-hydroxybutyl (*R*)-3-hydroxybutyrate (HVMN Inc., San Francisco, CA, USA). The isocaloric, dextrose placebo beverage was prepared by mixing dextrose powder in water. To mask the differences in taste, bitterness, and color between the KET and DEX beverages, zero-calorie stevia sweetener (SweetLeaf Sweetener; Wisdom Natural Brands, Gilbert, AZ, USA) and lemon- and cranberry raspberry-flavored Mio (Kraft Foods; H.J. Heinz Company Brands LLC., Glenview, IL, USA) were added to both experimental beverages. All drinks were diluted to 250 mL with water. Participants wore a nose clip and immediately consumed 20 mL of Powerade Zero (Coca-Cola Ltd., Toronto, ON, Canada) following the KET or DEX drink based on methods previously described (15). Using this protocol, pilot testing confirmed that research participants were not able to discern differences in the experimental beverages, however the success of the blinding was not directly verified amongst participants in this study.

Venipuncture was completed at baseline, 60, 90, and 150 min post-initiation of exercise. A validated, paper-based, 100 mm appetite visual analog scale (VAS) (16) was used to assess subjective measures of hunger, satisfaction, fullness, and prospective food consumption (PFC) at approximately the same time that each of the 4 venipuncture samples were collected, with an additional measure following the *ad libitum* meal. Further, finger capillary punctures were completed using a commercially available, contact-activated, 21G, 1.8 mm depth lancet (BD, Becton, Dickinson and Company, Mississauga, ON, Canada) at 7 time points throughout each condition (baseline, 30, 60, 90, 120, 150, and 180 min post-initiation of exercise) to measure blood βHB concentrations using β-ketone test strips (FreeStyle Precision Blood β-Ketone Test Strips; Abbott Laboratories, Saint-Laurent, QC, Canada) and a ketone monitoring system (FreeStyle Precision Neo; Abbott Laboratories).

Following the final blood draw (~90 min post-exercise) and the 150 min appetite VAS, participants consumed a pre-weighed *ad libitum* meal. This approach for measuring energy intake has been previously validated and verified as reproducible in males and females (13, 17). The meal consisted of bottled water (Nestle, Toronto, ON, Canada), 2% milk (Saputo, Montreal, QC, Canada), and Michelina's Signature Chicken Teriyaki (Bellisio Foods, Inc., Duluth, MN, USA). Prior to consenting to participate in the study, all participants indicated that they had no allergies or aversion to the selected food items. All meals were provided in surplus and served in an isolated, distraction free room. Participants were asked to consume the meal until comfortably full and were provided with 30 min to finish the meal. Post-meal, all remaining food was weighed using a digital scale to assess energy intake.

### Blood Collection and Analysis

All blood samples were collected into pre-cooled 6 mL K_2_EDTA spray-coated vacutainers (BD, Mississauga, ON, Canada). Immediately after collection, a protease inhibitor cocktail containing DPP IV inhibitor (10 μL·mL^−1^ blood; MilliporeSigma Corp., ON, Canada), Sigma protease inhibitor (1 mg·mL^−1^ blood; SigmaFast, MilliporeSigma Corp.), and Pefabloc (1 mg·mL^−1^ blood; MilliporeSigma Corp.) was added to the sample to prevent degradation of satiety hormones. Blood samples were centrifuged at 2,500 g for 10-min at 4°C. Plasma aliquots were stored at −80°C for later analysis.

The concentration of PYY was determined using the Human PYY (Total) ELISA kit (MilliporeSigma Corp.). GLP-1 concentration was assessed using the High Sensitivity GLP-1 Active Chemiluminescent ELISA kit (MilliporeSigma Corp.). Acyl-ghrelin concentration was assessed by the Human Ghrelin (Active) kit (MilliporeSigma Corp). All samples were assayed in duplicate. The intra- and inter-assay variation for these assays ranges between 2 and 7% in our laboratory, as previously described (18).

### Statistical Analysis

SPSS software version 26 was used to analyze the data. Our primary outcome measures were appetite-regulating hormone changes following the exercise session. Sample size estimations were completed using G^*^Power (α = 5%, β = 80%) using an effect size of 0.75 and the analysis of variance (ANOVA): repeated measures, between factors statistical test. The effect size was based on previously published research showing reductions in both GLP-1 and ghrelin with KE (5, 15) as well as previously determined standard deviation values from the measurement of these hormones in our lab (18). Data was assessed for normality using the Shapiro–Wilk test. Differences in perceived appetite, βHB, acyl-ghrelin, GLP-1, and PYY were assessed using two-way repeated measures ANOVA. When there was a significant condition × time interaction, *post-hoc* least significant difference pairwise comparisons were used to determine differences at specific time points. Within-condition differences were examined using a one-way repeated measures ANOVA. Effect size (*d*) was calculated using Cohen's d. Area under the curve (AUC) analysis was completed for hormonal analysis and appetite perceptions. AUC estimations were calculated using trapezoidal sums. Correlation analysis was calculated between energy intake and AUC for acyl-ghrelin, GLP-1, and PYY using a two-tailed Pearson test. Statistical significance was set at *P* ≤ 0.05. All data in the manuscript are represented as mean ± SD.

## Results

### Participants

Thirteen participants completed the study (7 female, 6 male). Participants were on average 23.6 ± 2.4 years of age, weighed 73.7 ± 10.9 kg and had a body mass index of 25.7 ± 3.2 kg·m^−2^ (Table 1). Average VO_2peak_ was 37.6 ± 4.3 mL O_2_·kg^−1^·min^−1^.

**Table 1:** Participant characteristics (*n* = 13; 7 female, 6 male).

	**mean ± SD (range)**
Age (years)	23.6 ± 2.4 (21–30)
Height (m)	1.69 ± 0.06 (1.59–1.82)
Weight (kg)	73.7 ± 10.9 (59.0–91.2)
BMI (kg·m^−2^)	25.7 ± 3.2 (21.2–30.0)
VO_2_peak (mL O_2_·kg^−1^·min^−1^)	37.6 ± 4.3 (30.3–47.9)

### Blood βHB Levels

Blood βHB was measured seven times (baseline, 30, 60, 90, 120, 150, and 180 min after the initiation of exercise) throughout each condition. A significant interaction effect of condition × time (*P* < 0.001), a main effect of condition (*P* < 0.001), and a main effect of time (*P* < 0.001) were observed for blood βHB levels. *Post-hoc* comparisons revealed higher concentrations of blood βHB in the KET condition compared to DEX at 30 (*P* < 0.001; *d* = 3.23), 60 (*P* < 0.001; *d* = 3.59), 90 (*P* < 0.001; *d* = 6.23), 120 (*P* < 0.001; *d* = 10.47), 150 (*P* < 0.001; *d* = 7.85), and 180 (*P* < 0.001; *d* = 6.71) min following the initiation of exercise. No differences in baseline measures of βHB (*P* = 0.111; *d* = 0.48) were observed between the KET and DEX conditions. Elevated ketonaemia was achieved with the pre-exercise 0.5 g·kg^−1^ KE dose, with βHB concentrations reaching ~2.9 mM (range 1.2-4.5 mM) (Figure 1B). After the additional post-exercise 0.25 g·kg^−1^ KE, βHB concentrations were elevated to ~5.0 mM (range 4.1–6.1 mM). The isocaloric dextrose drink had no impact on blood βHB levels (Figure 1B). With a total dose of 0.75 g·kg^−1^ KE, participants received an average of 265 kcal (~4.8 kcal·g^−1^ KE). To create an isocaloric, dextrose-containing control beverage, participants received an average of 66 g total dextrose.

### Appetite-Related Hormones

#### Acyl-Ghrelin

**Figure 2:**
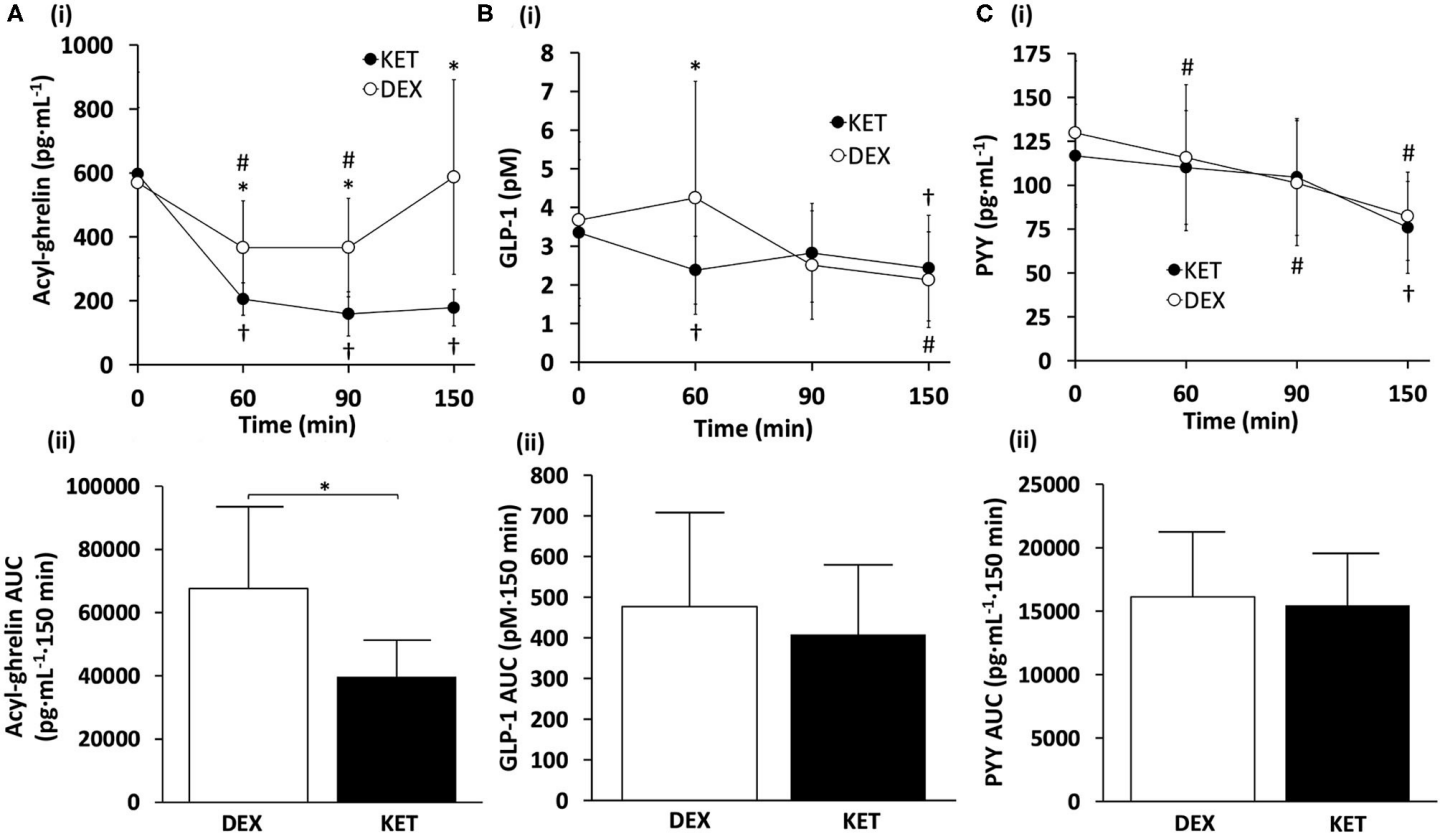
(i) Hormone concentrations and (ii) AUC measures of **(A)** acyl-ghrelin, **(B)** GLP-1, and **(C)** PYY throughout the KET and DEX conditions. Values are means ± SD, *n* = 13. *P < 0.05 between KET and DEX; ^†^*P* < 0.05 KET within-condition relative to baseline; ^#^*P* < 0.05 DEX within-condition relative to baseline. DEX, dextrose condition; GLP-1, glucagon-like peptide-1; KET, ketone condition; PYY, peptide tyrosine tyrosine.

There was a significant condition × time interaction (*P* < 0.001), main effect of condition (*P* = 0.001), and main effect of time (*P* < 0.001) for acyl-ghrelin (Figure 2A). *Post-hoc* pairwise comparisons revealed that the KET condition had lower acyl-ghrelin compared to DEX at 60 min (*P* = 0.002; *d* = 1.12), 90 min (*P* = 0.002; *d* = 1.06), and 150 min (*P* = 0.001; *d* = 1.30) following the initiation of exercise. No differences in acyl-ghrelin concentrations were observed between conditions at baseline (*P* = 0.622; *d* = 0.14). Within-condition, acyl-ghrelin was reduced at 60 min (*P* = 0.001), 90 min (*P* < 0.001), and 150 min (*P* < 0.001) relative to baseline in the KET condition. In the DEX condition, acyl-ghrelin was reduced only at 60 min (*P* = 0.003) and 90 min (*P* = 0.004) relative to baseline. AUC comparisons revealed lower concentrations of acyl-ghrelin (*P* = 0.001; *d* = 1.15) during the KET condition compared to DEX.

#### Glucagon-Like Peptide-1 (GLP-1)

For GLP-1, there was a significant condition × time interaction (*P* = 0.026) and a main effect of time (*P* = 0.007) (Figure 2B). No main effect of condition was observed (*P* = 0.290). *Post-hoc* comparisons for GLP-1 showed lower blood concentrations of GLP-1 in the KET condition at 60 min (*P* = 0.038; *d* = 0.65) relative to DEX. Between conditions, no differences were observed in GLP-1 at baseline (*P* = 0.567; *d* = 0.16), 90 min (*P* = 0.366; *d* = 0.26), or 150 min (*P* = 0.286; *d* = 0.31). Within-condition, GLP-1 in the KET condition was suppressed immediately after exercise, relative to baseline values (*P* = 0.03), and at 150 min for both DEX (*P* = 0.014) and KET (*P* = 0.023) conditions. No AUC differences were observed between conditions for GLP-1 (*P* = 0.221; *d* = 0.36).

#### Peptide Tyrosine Tyrosine (PYY)

No significant interaction of condition × time (*P* = 0.228) or main effect of condition (*P* = 0.574) were observed for PYY (Figure 2C). However, there was a significant main effect of time for PYY (*P* < 0.001). No differences were observed between conditions for baseline concentrations of PYY (*P* = 0.166; *d* = 0.41). Within-condition, PYY decreased at 60 min (*P* = 0.030) and 90 min (*P* = 0.007) with DEX, and 150 min for both DEX (*P* < 0.001) and KET (*P* < 0.001), in relation to baseline measures. There were no differences between conditions for AUC PYY (*P* = 0.654; *d* = 0.13).

### Perceived Appetite

**Figure 3:**
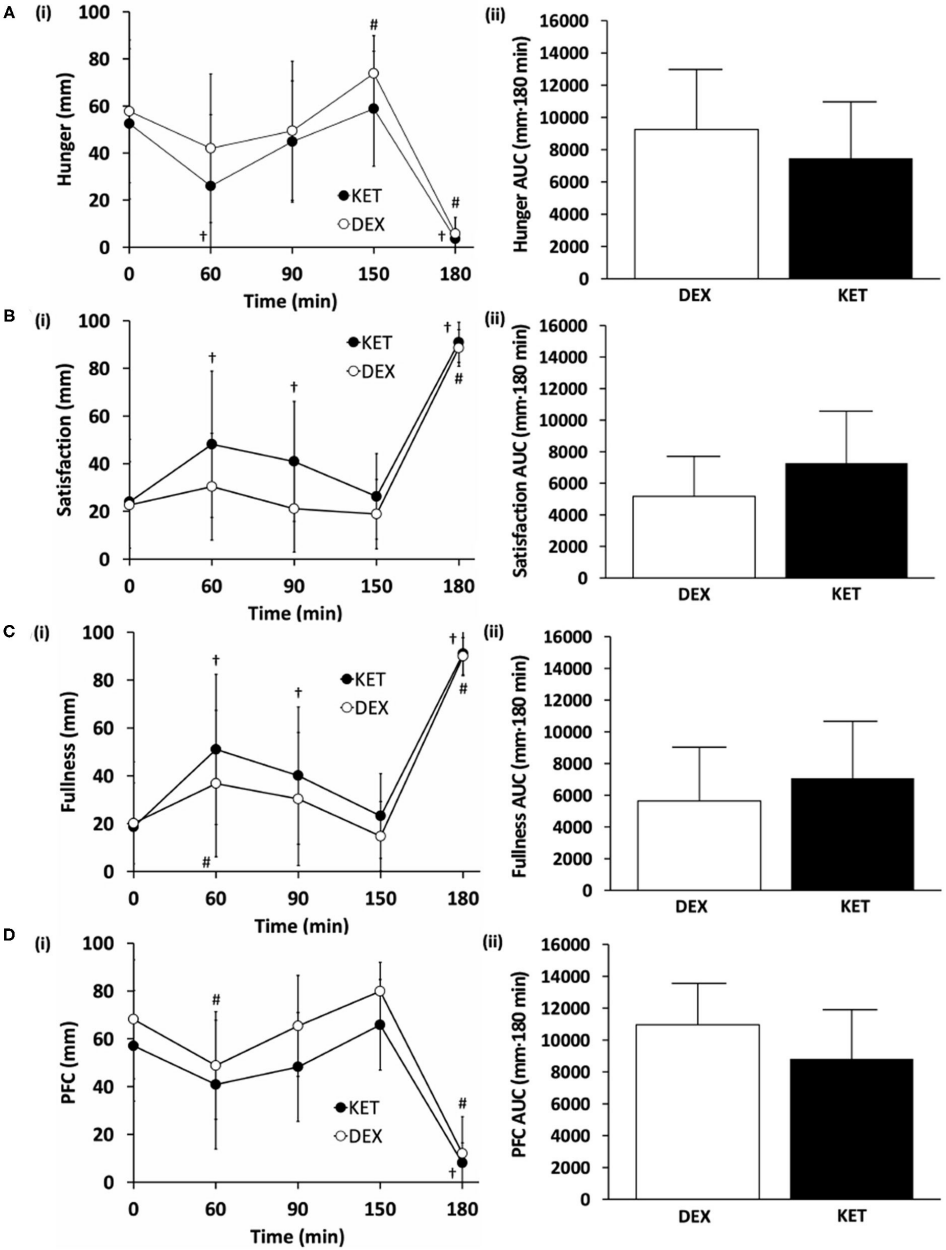
Subjective measures of **(A)** hunger, **(B)** satisfaction, **(C)** fullness, and **(D)** prospective food consumption (PFC) on a (i) 100 mm visual analog scale (VAS) and (ii) total area under the curve (AUC) between the KET and DEX conditions. Values are means ± SD, *n* = 13. ^†^*P* < 0.05 KET within-condition relative to baseline. ^#^*P* < 0.05 DEX within-condition relative to baseline. DEX, dextrose condition; KET, ketone condition; PFC, prospective food consumption; VAS, visual analog scale.

Levels of subjective hunger, satisfaction, fullness, and PFC were measured at 5 time points (baseline, 60, 90, 150, and 180 min following the initiation of exercise). No condition × time interactions (hunger, *P* = 0.388; satisfaction, *P* = 0.082; fullness, *P* = 0.282; PFC, *P* = 0.254) or main effects of condition (hunger, *P* = 0.161; satisfaction, *P* = 0.65; fullness, *P* = 0.279; PFC, *P* = 0.083; fullness, *P* = 0.279) were observed (Figure 3). There was a main effect of time for all appetite ratings (*P* < 0.001). There were no differences in perceived appetite measures at baseline (hunger, *P* = 0.620, *d* = 0.14; satisfaction, *P* = 0.862, *d* = 0.04; fullness, *P* = 0.860, *d* = 0.05; PFC, *P* = 0.196, *d* = 0.52). AUC comparisons between KET and DEX showed a trend toward increased satisfaction with KET (*P* = 0.057; *d* = 0.59). No differences in AUC were seen for feelings of hunger (*P* = 0.154; *d* = 0.44), fullness (*P* = 0.239; *d* = 0.34), or PFC (*P* = 0.084; *d* = 0.52).

### Energy Intake

**Figure 4:**
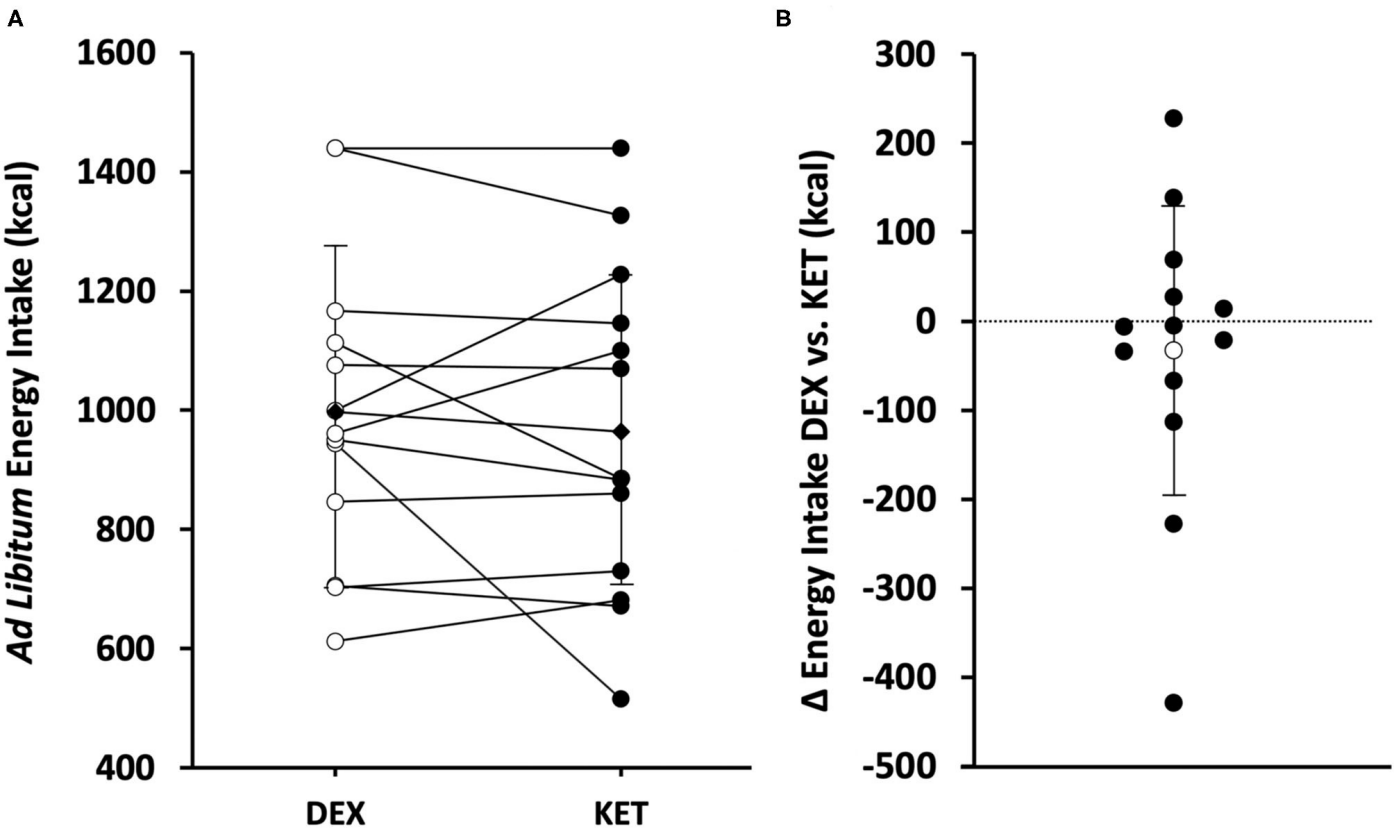
**(A)** Individual (white circle = DEX; black circle = KET) and average (diamond) *ad libitum* energy intake (kcal) in the KET vs. DEX conditions. **(B)** Individual (black circle) and average (white circle) Δ in *ad libitum* energy intake from DEX to KET conditions. Values are means ± SD, *n* = 13. DEX, dextrose condition; KET, ketone condition.

*Ad libitum* energy intake was measured 150 min following the initiation of exercise (90 min post-exercise). There were no differences in energy intake (*P* = 0.488; *d* = 0.20) between KET (964 ± 279 kcal; range 515–1,440 kcal) and DEX (997 ± 257 kcal; range 612–1,440 kcal) (Figure 4). No correlations were observed between energy intake and acyl-ghrelin (*r* = 0.226; *P* = 0.266), GLP-1 (*r* = −0.015; *P* = 0.941), and PYY (*r* = −0.103; *P* = 0.617).

## Discussion

This study examined the impact of exogenous ketosis during and after a bout of aerobic exercise on homeostatic regulators of appetite and *ad libitum* energy intake. Participants received a cumulative dose of 0.75 g·kg^−1^ KE and increased blood βHB concentrations to 4.1–6.1 mM during the post-exercise period. Ketosis, in association with exercise, reduced the hunger hormone acyl-ghrelin as well as the satiety hormone GLP-1. Post-exercise perceived appetite and *ad libitum* energy intake were not affected by exogenous ketosis. While βHB transiently modifies the secretion of appetite-regulating hormones following exercise, these changes do not appear to acutely affect subjective appetite and post-exercise energy intake/compensation.

Numerous studies have demonstrated that nutritionally-induced ketosis attenuates the increase in the hunger hormone ghrelin that occurs in association with weight loss (19–21). Similarly, acute exogenous ketosis under sedentary and exercise conditions has also been shown to induce transient reductions in acyl-ghrelin (5, 11). Consistent with these studies, we show a reduction in acyl-ghrelin with exogenous ketosis when compared to the consumption of an isocaloric dextrose beverage. Within-condition, both the ketone monoester and dextrose led to reductions in acyl-ghrelin at 60 min compared to baseline levels. The reduction in acyl-ghrelin in the DEX group is likely attributable to the combined impact of exercise and carbohydrate ingestion, both of which lead to transient reductions in acyl-ghrelin (18). Despite the absence of carbohydrate in the ketone ester group, the further reduction in acyl-ghrelin suggests that βHB elicits greater inhibitory action on acyl-ghrelin than carbohydrate alone. While many studies demonstrate an inverse association between βHB and acyl-ghrelin, the mechanism by which βHB reduces acyl-ghrelin is not completely understood. Acyl-ghrelin is produced in oxyntic cells in the stomach and the secretion of ghrelin is believed to be stimulated by the catecholamine-induced activation of β1-adrenergic receptors (22, 23). Interestingly, βHB has been demonstrated to suppress sympathetic tone by antagonizing GPR41 receptors expressed on sympathetic ganglion (24). It is not clear, however, whether sympathetic tone is reduced under exercise conditions with exogenous ketosis (11). Altogether, there is consistent evidence showing a reduction in acyl-ghrelin with KE ingestion, however the mechanisms mediating this reduction have yet to be fully elucidated.

In contrast to the anorexigenic effects of reduced acyl-ghrelin, the gut-derived satiety hormone GLP-1 was reduced post-exercise with ketone ester ingestion. Exercise, alone and in combination with nutrient intake, is known to lead to short-term increases in GLP-1 (8, 18). In two previous studies, ketone esters acutely suppressed GLP-1 under sedentary conditions (5, 15). Given that dextrose is known to induce GLP-1 secretion (25), it is possible that the reduction in GLP-1 in the ketone ester group is due to a lack of dextrose-induced GLP-1 secretion. Evidence from Myette-Côté et al. (15), however, shows that ketone esters acutely suppress GLP-1 even when carbohydrate intake is the same between experimental groups, suggesting that βHB may have inhibitory action on GLP-1 secretion. βHB has antagonizing action on GPR41 receptors (24), the same receptors believed to be responsible for gut-stimulated GLP-1 secretion. In mice lacking the GPR41 receptor, short chain fatty acid-induced GLP-1 secretion is blunted (26). Based on this evidence, βHB may inhibit GLP-1 secretion by blocking GPR41 receptors on enteroendocrine cells within the gut. Overall, despite an unclear mechanism, the evidence suggests that acute ketosis elicits reductions in GLP-1, a finding that stands in contrast to the purported anorexigenic effects associated with ketosis.

Although numerous studies have examined changes in appetite signals with exogenous ketosis, there are no studies that have examined the effects of acute ketone ester supplementation on *ad libitum* energy intake in humans. Here we report that elevated ketone concentrations after an exercise session yield no changes in *ad libitum* energy intake. This finding is consistent with the observation that perceived appetite was not affected by ketone ingestion. Stubbs et al. (5) have previously reported that exogenous ketosis lowers subjective hunger and desire to eat under sedentary conditions. Exercise alone is known to reduce post-exercise appetite (27). The failure to see a reduction in perceived appetite in our study may be due to a greater anorexigenic effect of acute exercise in relation to exogenous ketosis. Despite this, under intense exercise conditions, ketosis has been found to lower post-exercise perceived appetite (11). After a 3 h intermittent cycling session, 15 min time trial, and an all-out sprint, Poffe et al. observed reductions in perceived levels of hunger and desire to eat when the exercise session was completed in a state of ketosis. Relative to our study findings, the reductions in perceived appetite reported by Poffe et al. may be due to factors including exercise duration/intensity, differential state of ketosis at the time of perceived appetite assessment, or additional energy consumption associated with exogenous ketone ingestion (~277 kcal in the ketone condition relative to control) (11). In response to prolonged exogenous ketosis, animal studies show that ketones can reduce absolute energy intake (6, 28–30). Dietary R-3-hydroxybutyrate-R-1,3-butanediol monoester (~30% energy) provided to C57BL/6J mice for a period of 12 weeks resulted in a 26% reduction in energy intake (28). Deemer et al. (6) showed that a diet with ~30% of energy from ketone esters is necessary to induce this reduction in energy intake. It remains unknown, however, whether olfactory and taste aversion factors with ketone supplementation affects energy intake in animal studies (28). In contrast to prolonged animal studies showing reductions in energy intake with exogenous ketosis, it has been also been reported that ketone ester supplementation for a period of 3 weeks (50–57 g KE, 6×/week for 3 weeks) can increase energy intake in athletes performing high volumes of physical activity (31). It is speculated that the elevation in energy intake in a ketotic state is mediated by a reduction in the satiety signal growth differentiation factor 15. Potentially, given that ketone bodies cannot be converted into a storage form of energy (e.g., triglyceride) and are excreted in urine and breath if not oxidized (32), it is possible that ketone excretion elicits a greater energy deficit relative to isocaloric macronutrient ingestion, thus contributing to a greater compensatory response in energy intake. Taken together, the influence of exogenous ketosis on energy intake remains unclear. Under exercise conditions, our study suggests that acute ketosis has a neutral influence on energy intake.

There are several limitations with this present study. Our study did not include a non-exercise, sedentary control group. As such, it was not possible to determine whether there were any interaction effects between exercise and ketone ester intake on measures of appetite-regulating hormones, perceived appetite, or *ad libitum* energy intake. Additionally, our study may not have been adequately powered to detect differences in perceived appetite and energy intake. Although not statistically significant, there was a consistent pattern for exogenous ketosis to lower perceived appetite and energy intake, with effect sizes ranging from 0.2 to 0.6. Furthermore, given that this study was conducted in individuals with normal weight and average aerobic fitness, the findings from our study cannot be generalized to a population with obesity or high-performance aerobic athletes. Lastly, it was not possible to conduct the *ad libitum* meal tests using food items regularly consumed by all research participants. It is possible that the selected food items, unfamiliar eating environment, and restrictions on the timing of the *ad libitum* meal may have influenced energy intake measures.

In conclusion, our study provides evidence that acute exogenous ketosis during and after exercise elicits opposing action on homeostatic regulators of appetite by lowering the gut-derived, orexigenic hormone acyl-ghrelin as well as the anorexigenic hormone GLP-1. Associated with these changes, exogenous ketosis did not affect post-exercise appetite or alter *ad libitum* energy intake. In healthy adults, these data suggest that acute ketosis, in conjunction with a 1 h moderate to vigorous intensity aerobic exercise session, is not likely to have a large influence on short-term, post-exercise energy balance.

## Data Availability Statement

The raw data supporting the conclusions of this article will be made available by the authors, without undue reservation.

## Ethics Statement

The studies involving human participants were reviewed and approved by University of Lethbridge Human Participant Research Committee. The patients/participants provided their written informed consent to participate in this study.

## Author Contributions

TO recruited participants, collected and analyzed data, and wrote the manuscript. TQ recruited participants and collected data. MB designed study, obtained funding, and had final responsibility for the study. All authors had access to the study data and reviewed and approved the final manuscript.

## Conflict of Interest

The authors declare that the research was conducted in the absence of any commercial or financial relationships that could be construed as a potential conflict of interest.
